# The ribbon-associated protein C-terminal-binding protein 1 is not essential for the structure and function of retinal ribbon synapses

**Published:** 2013-04-18

**Authors:** Thirumalini Vaithianathan, Wendy Akmentin, Diane Henry, Gary Matthews

**Affiliations:** 1 Department of Neurobiology and Behavior, Stony Brook University, Stony Brook, NY; 2 SUNY Eye Institute, Stony Brook University, Stony Brook, NY; 3 Department of Ophthalmology, Stony Brook University, Stony Brook, NY

## Abstract

**Purpose:**

Synaptic ribbons are organelles found at presynaptic active zones of sensory neurons that generate sustained graded electrical signals in response to stimuli, including retinal photoreceptor cells and bipolar neurons. RIBEYE is the major and specific protein constituent of ribbons; however, over the past decade an increasing number of other proteins have been identified at ribbon active zones, including C-terminal-binding protein 1 (CtBP1; a regulator of transcription and membrane trafficking that might bind to the B domain of RIBEYE). The appearance of CtBP1 together with RIBEYE suggests that it may contribute to ribbon function, but the possible role of CtBP1 at ribbon synapses has not yet been examined. Using CtBP1-knockout mice, we tested for functional effects of absence of CtBP1 protein.

**Methods:**

Confocal microscopy, electrophysiology, and electron microscopy were used to examine the structure and function of ribbon synapses in the retina and in isolated bipolar neurons from CtBP1 null mice compared with their wild-type littermates.

**Results:**

Expression of ribbons appeared to be normal in CtBP1 null mouse retina as revealed by immunofluorescence with an antibody to the B domain of RIBEYE and by binding studies using a fluorescent peptide that binds to RIBEYE in ribbons of living bipolar cells. Electron microscopy also showed grossly normal pre- and postsynaptic organization of ribbon synapses in both photoreceptors and bipolar cells. Synaptic vesicles were normal in size, but the overall density of reserve vesicles was reduced by ~20% in the cytoplasm of CtBP1 null ribbon synaptic terminals. However, the reduced vesicle density did not detectably alter synaptic function of bipolar neurons as revealed by activity-dependent loading of synaptic vesicles with FM4–64, presynaptic calcium current, capacitance measurements of synaptic exocytosis, and destaining of FM dye upon stimulation.

**Conclusions:**

Overall the results suggest that CtBP1 protein is not essential for the formation of functional ribbon synapses in the retina.

## Introduction

Visual and auditory sensory neurons are capable of tonic release of neurotransmitter, and their presynaptic active zones have a specialized organelle, called the synaptic ribbon, that tethers numerous synaptic vesicles that are thought to support continuous release. The major structural protein of synaptic ribbons, RIBEYE, is derived from the gene encoding the transcriptional cofactor C-terminal-binding protein 2 (CtBP2) by use of an alternative promoter and first exon [1]. The first 20 amino acids of CtBP2 are replaced in RIBEYE by a structural A domain, but the remainder of CtBP2 is retained as the B domain of RIBEYE. In addition, the related transcriptional cofactor CtBP1 is also associated with ribbons [2]. Since CtBPs are known to form heterodimers [3,4], CtBP1 may be recruited to ribbons by directly binding to the B domain of RIBEYE. RIBEYE is critical for ribbon assembly and function [5-7], but the functional importance of CtBP1 at ribbons is unknown. A splice variant of CtBP1 has been implicated in regulating membrane fission in the Golgi complex [8], which raises the question of whether CtBP1 may also play a role in vesicle trafficking at ribbon synapses. To examine the possible role of CtBP1 in retinal ribbon synapses, we compared ribbon structure and function in CtBP1 null mice [9] and in their wild-type (WT) littermates. We found that the expression, ultrastructure, and function of ribbon synapses were not significantly affected by the absence of CtBP1.

## Methods

### Transgenic animals

A breeding pair of mice heterozygous for a null mutation in *CtBP1* (strain B6;129S4-*Ctbp1^tm1Sor^*/J) was purchased from Jackson Laboratory (Bar Harbor, ME) and used to start a colony at the State University of New York, Stony Brook. Genotypes of offspring were determined by PCR by using 100 ng of genomic DNA and pairs of primers that amplify the mutant or the WT allele. Primer pair 1 (forward, 5'-GAA GTA CCA GTA CAG GGG ACG-3'; reverse, 5'-CCC CAG CTG ACT TGA TGT CG-3') amplifies a 568-bp fragment from the WT *CtBP1* gene, and primer pair 2 (forward, 5'-CGG TCT TGT CGA TCA GGA TGA TCT GG-3'; reverse, 5'-CAG CCC ATT CGC CGC CAA GC-3') amplifies a 263-bp product from the inserted transgene. After denaturation for 3 min at 94 °C, reactions were performed for 35 cycles of 30 s at 94 °C, 30 s at 56 °C, and 30 s at 72 °C. Experiments were performed using CtBP1^−/−^ mice (knockout [KO] mice) and their CtBP1^+/+^ littermates (WT mice).

### Preparation of isolated bipolar neurons

All procedures were approved by the Institutional Animal Care and Use Committee of the State University of New York at Stony Brook and followed guidelines of the US Public Health Service. A mouse was killed by CO_2_ inhalation, and both eyes were removed and hemisected. The procedure for acutely isolating bipolar cells from mouse retina was similar to that described previously [10]. Briefly, retinas were dissected from eyecups in standard solution, (NaCl 135 mM, KCl 5 mM, CaCl_2_ 0.5 mM, MgCl_2_ 1 mM, HEPES 10 mM, pH 7.4), treated for 25 min with hyaluronidase (500 units/ml; Worthington Biochemical, Lakewood, NJ), and then transferred to cold saline containing 2.7 mM DL-cysteine (Sigma-Aldrich, St. Louis, MO), cut into small pieces, and incubated 25–30 min in saline containing DL-cysteine and 15–30 units/ml papain (Sigma) at room temperature. A piece of retina was triturated via a flame-polished Pasteur pipette, and dissociated cells were plated onto glass-bottomed dishes containing saline with CaCl_2_ increased to 2.5 mM for patch-clamp recording. Isolated bipolar cells were identified by their characteristic morphology [11].

### Electrophysiology and activity-dependent loading with fluorescent dye

Whole-cell patch-clamp recordings were made from acutely dissociated rod bipolar cells with the patch pipette placed directly on the synaptic terminal. Rod bipolar cells were identified on the basis of their characteristic shape, that is, a spheroid soma of 5–8 μm in diameter and a relatively stout axon and a cluster of lobulated terminal endings of ~3 μm diameter [11-14]. The pipette solution contained: Cs-gluconate 120 mM, tetraethylammonium chloride 10 mM, MgCl_2_ 3 mM, N-methyl-d-glucamine (NMDG)-EGTA 0.2 mM, Na_2_ATP 2 mM, sodium guanosine-5'-triphosphate (Na_2_GTP) 0.5 mM, HEPES 20 mM, pH 7.2. Prior to recording, bipolar cell terminals were loaded with FM4–64 (Life Technologies, Grand Island, NY) by exchanging the bath solution with a high-K+ solution (80 mM KCl replacing NaCl) + 5 µM FM4–64 for 90 s, followed by multiple washes with saline, 60 s in standard solution + 1 mM Advasep-7 (Biotium, Hayward, CA), and again multiple washes with standard solution. Bipolar cells were then selected for recording based on good viability, intact morphology, and FM4–64 uptake restricted to synaptic terminals. Membrane currents were recorded under voltage-clamp using a HEKA EPC-9 amplifier controlled by PatchMaster software (HEKA, Bellmore, NY). Holding potential was −60 or −65 mV, and calcium channels were activated by pulses of varying duration to 0 mV. Membrane capacitance, series conductance, and membrane conductance were measured using the sine+DC method of the PatchMaster lock-in extension and a 1600-Hz sinusoidal stimulus with peak-to-peak amplitude of 10 mV centered on the holding potential. Pipettes were coated with dental wax to reduce stray capacitance.

### Fluorescence imaging

Fluorescence images were acquired using an Olympus FV-1000 laser-scanning confocal system connected to an Olympus IX-81 inverted microscope, controlled by Olympus FV10-ASW software (Olympus Imaging America Inc., Center Valley, PA). For live-cell imaging, care was taken to minimize photobleaching and phototoxicity by using a fast scan speed (2 µs/pixel), low laser intensity (0.1%–0.5% of maximum), and low pixel density (frame size 64×64 to 256×256 pixels). Images were analyzed using ImageJ (imagej.nih.gov). Immunofluorescence staining of cryosections of mouse retina was performed as described previously [15]. In brief, sections were incubated overnight at 4° C with mouse monoclonal antibody against CtBP2 (catalog number 612044, 1:5000 dilution; BD Transduction Laboratories, BD Biosciences, San Jose, CA) or mouse monoclonal antibody against CtBP1 (catalog number 612042, 1:5000 dilution; BD Transduction Laboratories, BD Biosciences), followed by goat anti-mouse secondary antibody labeled with Alexa Fluor 488 (catalog number A11029, 1:1000 dilution; Life Technologies). These primary antibodies are the same as those shown by Hildebrand and Soriano [9] to be specific for CtBP1 and CtBP2 based on loss of the appropriate bands in immunoblots from CtBP1 null and CtBP2 null embryonic stem (ES) cells. The CtBP2 antibody also detects RIBEYE [16], which is the main structural protein of ribbons that is also encoded by the gene for CtBP2. Therefore, this antibody was used to label retinal ribbons in immunofluorescence studies.

### Electron microscopy

Isolated retinas from four KO mice and four WT mice were fixed overnight at 4 °C in 2.5% paraformaldehyde + 2.5% glutaraldehyde in 0.1 M phosphate buffer (PB; pH=7.4), washed several times in 0.1 M PB, and postfixed in 1% osmium tetroxide in 0.1 M PB for 30 min. After washing and dehydration, retinas were embedded in Embed 812 resin (Electron Microscopy Sciences, Hatfield, PA) and sectioned on an ultramicrotome (Reichert-Jung, Buffalo, NY). Sections were then viewed and photographed using a JEOL 1200EX2 transmission electron microscope (JEOL USA, Inc., Peabody, MA).

### Statistical analysis

Statistical significance of differences in measured parameters between WT and CtBP1-KO samples was assessed using unpaired, two-tailed *t* tests with unequal variance.

## Results

### CtBP1 is not essential for the assembly of ribbons

**Figure 1:**
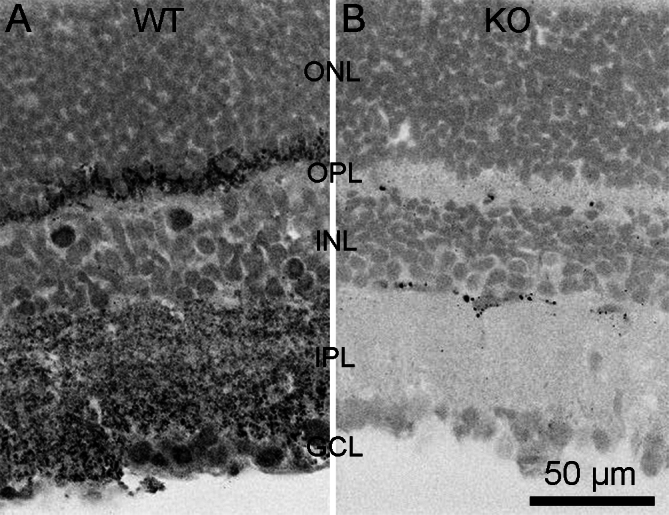
C-terminal-binding protein 1 (CtBP1) immunostaining at ribbon synapses is absent in CtBP1-knockout mouse retina. **A**: The panel shows an example of a confocal image obtained from a transverse section of WT mouse retina stained with a mouse monoclonal antibody against CtBP1. The intensity scale is inverted, so that brighter spots appear in black. ONL refers to the outer nuclear layer, OPL indicates the outer plexiform layer, INL denotes the inner nuclear layer, IPL points to the inner plexiform layer, and GCL specifies the ganglion cell layer. **B**: The panel shows an example of a confocal image obtained from a transverse section of CtBP1-knock out (KO) retina stained with anti-CtBP1. Intensity scale is inverted. Staining in the plexiform and nuclear layers was similar to that in sections stained with the anti-mouse secondary antibody alone, without anti-CtBP1, indicating the absence of CtBP1 in KO retina. The scattered dark spots (bright staining) were also present in sections stained with the secondary antibody alone and likely reflect blood vessels. Scale bar in **B** applies to **A** as well.

To determine if CtBP1 expression in the retina of the B6/129 hybrid mouse strain used here is similar to that reported previously [2], we immunostained cryosections of WT retina with the same anti-CtBP1 antibody used in the prior work. Figure 1A shows that the pattern of staining reproduced the previous results [2], with discrete staining in both the outer plexiform layer (OPL) and the inner plexiform layer (IPL) consistent with the localization of CtBP1 at synaptic ribbons demonstrated previously by immunoelectron microscopy [2]. Therefore, we confirm that CtBP1 is expressed at retinal ribbon synapses in WT mice. By contrast, anti-CtBP1 immunostaining was undetectable in either the OPL or IPL of retinas from CtBP1-KO littermates (Figure 1B), which indicates that CtBP1 protein is in fact absent in mice that are homozygous for the null mutation in CtBP1, as expected from prior work using this mutant strain [9].

**Figure 2:**
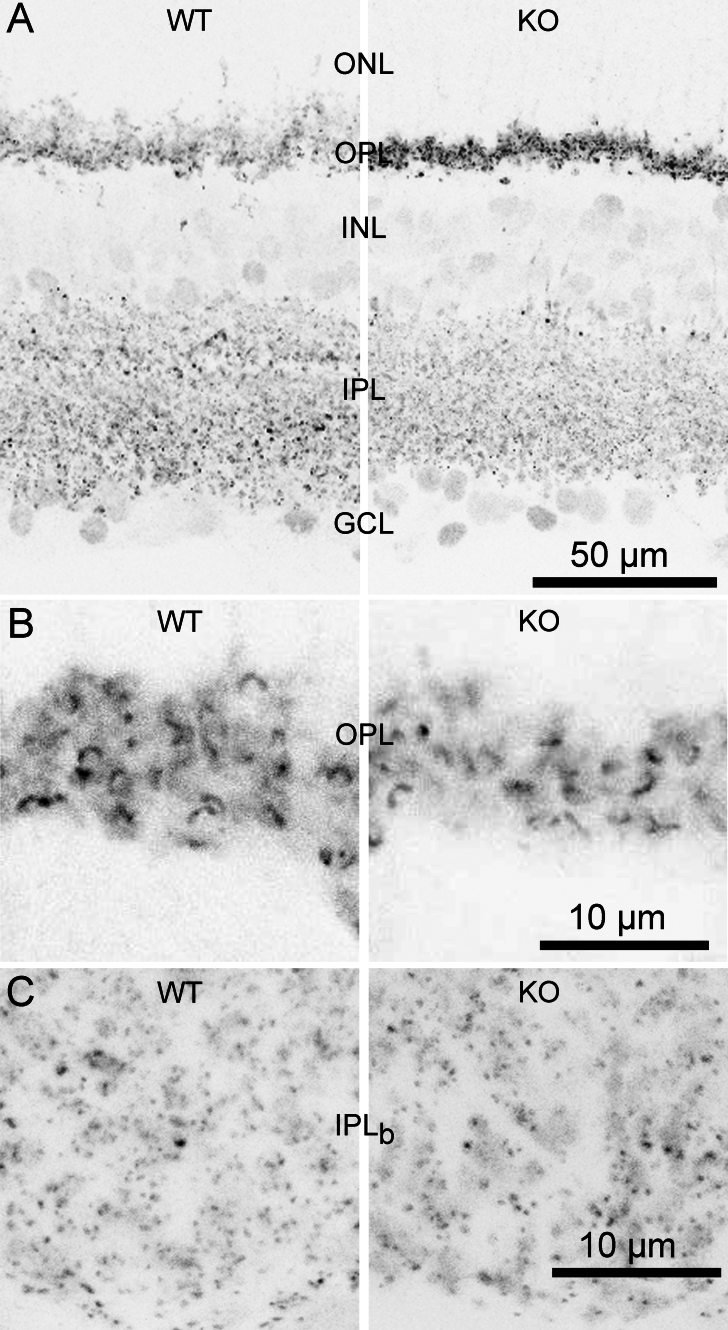
Overall morphology of the retina and localization of synaptic ribbons are normal in C-terminal-binding protein 1 (CtBP1)-knockout (KO) mice. **A**: The panel shows examples of confocal images obtained from transverse sections of wild-type (WT) and CtBP1-KO retina stained with an antibody against C-terminal-binding protein 2 (CtBP2) to reveal ribbons. The intensity scale is inverted for better visualization. ONL refers to the outer nuclear layer, OPL indicates the outer plexiform layer, INL denotes the inner nuclear layer, IPL points to the inner plexiform layer, and GCL specifies the ganglion cell layer. **B**: The panel shows a higher magnification confocal image of the OPL, containing the crescent-shaped ribbons of rod photoreceptors. **C**: The panel shows a higher magnification confocal image of sublamina b of the inner plexiform layer (IPL_b_), demonstrating the typical punctate ribbons of bipolar neurons.

Because CtBP1 is closely associated with the structural backbone of ribbons [2], we next asked whether ribbons form normally in the absence of CtBP1. To examine the overall expression of ribbons, we exploited the fact that ribbons are immunostained by an antibody against CtBP2 (e.g., [17,18]) because the antibody also binds to the ribbon protein RIBEYE whose B domain is identical to all but the N-terminal 20 residues of CtBP2 [1]. Figure 2 shows that staining of RIBEYE with anti-CtBP2 in the outer and inner plexiform layers was similar in WT and CtBP1-KO retina. In the OPL of both genotypes, RIBEYE staining had the distinctive crescent shape characteristic of the large ribbons of rod photoreceptors (Figure 2B), whereas staining in the IPL was dense and punctate (Figure 2C) as expected for the smaller more numerous ribbons of retinal bipolar neurons. Based on immunofluorescence staining, therefore, loss of CtBP1 apparently does not affect the presence of retinal ribbons.

**Figure 3:**
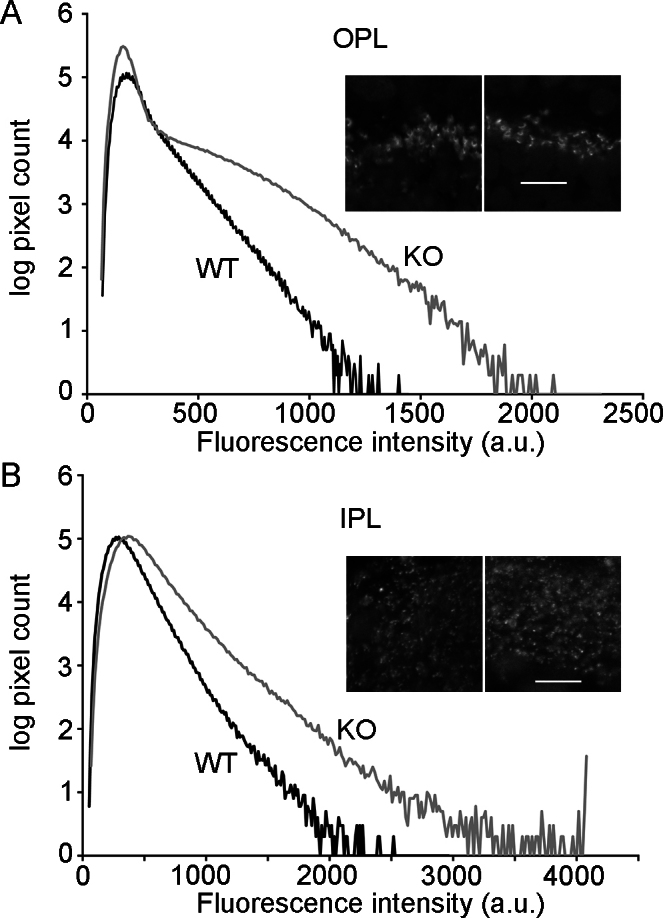
C-terminal-binding protein 2 (CtBP2) immunostaining at ribbons is brighter in C-terminal-binding protein 1 (CtBP1)-knockout retina. **A**: Histograms of pixel intensity were accumulated from a series of 868-μm^2^ images centered on the outer plexiform layer (OPL) of wild-type (WT; black trace) and CtBP1-knockout (KO; gray trace) retinas. The pixel count per bin (ordinate) is on a logarithmic scale. The WT histogram presents results from ten images. The KO histogram presents results from 13 images. The inset shows examples of individual images from WT (left) and KO (right) retina. The scale bar in the inset equals 10 μm. **B**: Histograms of pixel intensity were measured in the same way for the inner plexiform layer (IPL). WT and KO histograms present results from nine images. The inset shows examples of individual images from WT (left) and KO (right) retina. The scale bar in the inset equals 10 μm.

The fluorescence intensity of anti-CtBP2 staining at ribbons seemed to be higher in CtBP1-KO retina, which is particularly noticeable in the OPL-staining in Figure 2A. To make the comparison more carefully, cryosections of WT and KO retinas that were fixed in the same way were stained in parallel using the same primary and secondary antibody solutions; confocal images of the OPL and IPL were then acquired using identical settings. Histograms of pixel intensity measured using ImageJ for the OPL (Figure 3A) and IPL (Figure 3B) confirmed the visual impression that ribbon immunofluorescence was brighter in KO retina in both the OPL and IPL. One interpretation of this increased staining with anti-CtBP2 is that CtBP2 is localized at KO ribbons in the absence of CtBP1, adding to the immunofluorescence signal arising from staining of RIBEYE. This could arise via compensatory recruitment of cytoplasmic CtBP2 to the B domain of RIBEYE in CtBP1's stead (see Discussion).

**Figure 4:**
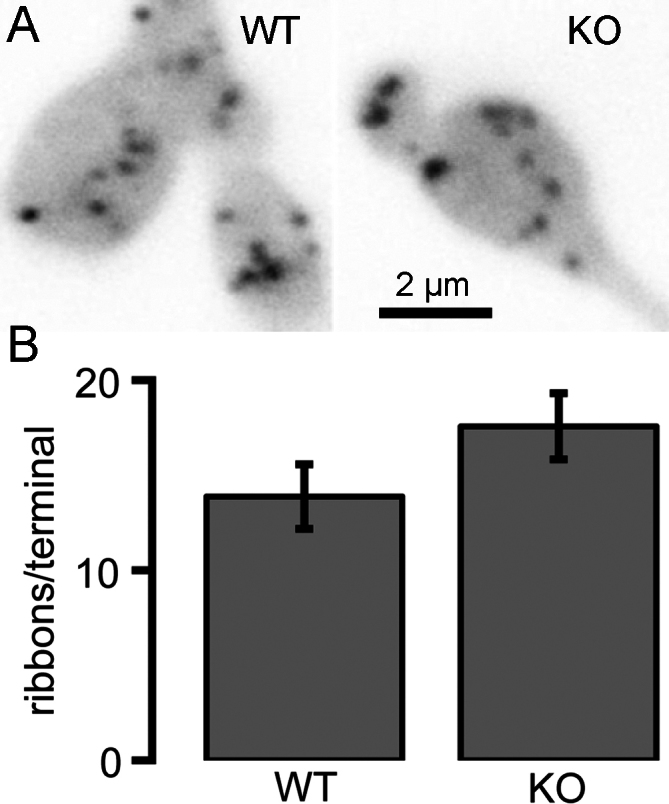
Isolated bipolar cells from wild-type and C-terminal-binding protein 1(CtBP1)-knockout retinas have similar numbers of ribbons marked with RIBEYE-binding peptide. **A**: The panels show confocal images of a live synaptic terminal loaded with RIBEYE-binding peptide in wild-type (WT; left) and CtBP1-knockout (KO; right) bipolar neurons. The peptide was dialyzed into cells via a whole-cell patch pipette. **B**: The average number of ribbons per terminal was not significantly different (p=0.22) between WT (n=10) and CtBP1-KO (n=11) bipolar neurons. The error bars indicate ±one standard error of the mean.

We also examined ribbons in individual bipolar neurons acutely isolated from CtBP1 KO and WT retinas by using live-cell imaging of a fluorescently labeled peptide that binds to RIBEYE [19] to identify ribbons. The terminal was dialyzed via a whole-cell patch pipette with 25 µM fluorescent RIBEYE-binding peptide (RBP; EQTVPVDLSVARPR), and the fluorescence of RBP rose rapidly with a time constant of ~6 s after break-in to enter whole-cell mode. Ribbons were then readily identified as bright fluorescent spots visible in confocal optical sections (Figure 4A), as described previously in goldfish and mouse bipolar cell terminals [10,19,20]. Ribbons were counted in a series of z-axis optical sections through the entire terminal, and the total number of ribbons was the same in the two genotypes (Figure 4B), averaging 13.9±1.7 (mean±standard error; n=8 cells in six animals) for WT terminals and 17.6±1.4 (n=7 cells in six animals) in CtBP1-KO terminals (p=0.22). This result at the level of individual bipolar neurons confirms the conclusion from immunostaining that ribbon expression is normal in CtBP1 null mice.

### Ultrastructure of ribbon synapses in the absence of CtBP1

**Figure 5:**
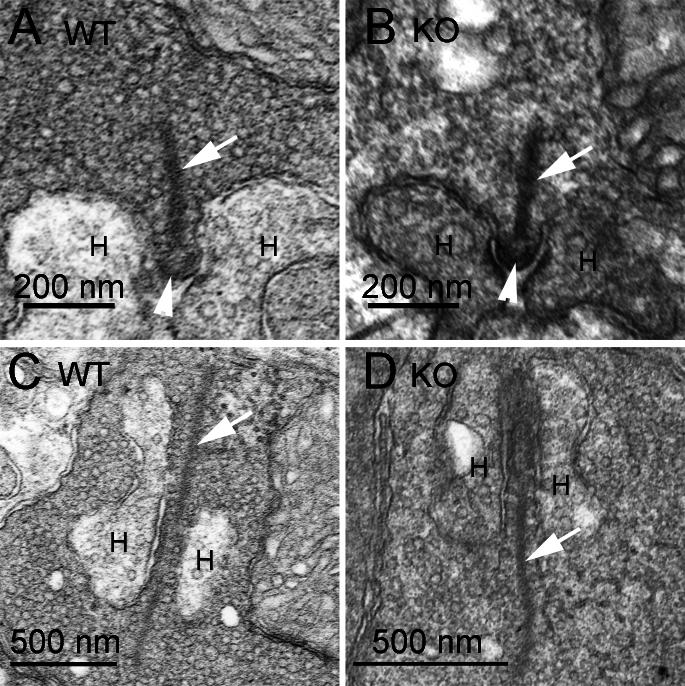
Normal ultrastructure of ribbons in the outer plexiform layer of C-terminal-binding protein 1 (CtBP1)-knockout retina. **A**, **B**: The electron micrographs show transverse sections through a rod ribbon synapse in the outer plexiform layer of wild-type (WT) and CtBP1-knockout (KO) retina. The arrow points to the ribbon, the arrowhead indicates the location of the arciform density, and the letter H marks the dendrites of horizontal cells. **C**, **D**: Electron micrographs of approximately horizontal sections through a rod ribbon synapse in WT and KO retina. The arrow indicates the ribbon, and the letter H marks the dendrites of horizontal cells.

**Figure 6:**
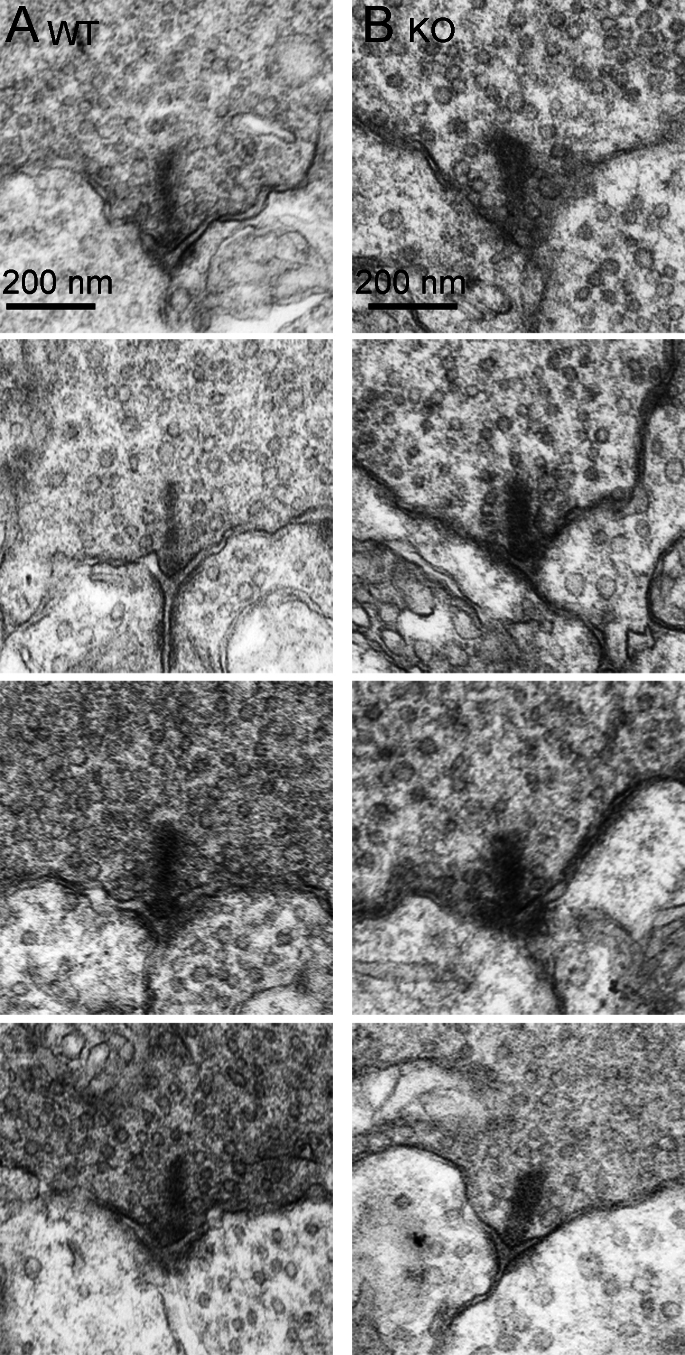
Normal ultrastructure of ribbons in the inner plexiform layer of C-terminal-binding protein 1 (CtBP1)-knockout retina. The electron micrographs show typical examples of ribbons in bipolar cells of wild-type (WT) retina (**A**) and CtBP1-knockout (KO) retina (**B**).

The observations using light microscopy of fixed tissue and living cells suggest that loss of CtBP1 does not grossly affect the formation of ribbons, but the data do not rule out more subtle effects on the structure of ribbons. Therefore, we used electron microscopy to examine the ultrastructure of ribbon synapses in WT and CtBP1-KO retinas. In the OPL of CtBP1-KO mice, ribbons of rod photoreceptors were indistinguishable from WT photoreceptors in both transverse (Figure 5A,B) and horizontal (Figure 5C,D) section planes, and the overall structure of the invaginating synapse was preserved with a pair of horizontal cell dendrites closely apposed to the ribbon at its attachment site with the plasma membrane of the active zone (Figure 5). In addition, the ultrastucture of ribbon synapses of bipolar neurons in the IPL was similar in the two genotypes (Figure 6); this was characterized by a small ribbon presynaptic to a pair of postsynaptic dendrites in both cases. Therefore, electron microscopy showed that ribbon synapses appeared to be ultrastructurally normal in both the OPL and IPL, despite the absence of CtBP1.

Because a splice variant of CtBP1 has been implicated in vesicle formation and membrane fission in other contexts [8,21-23], we also compared the size and density of synaptic vesicles at ribbon synapses of WT and CtBP1 mice. Synaptic vesicles associated with ribbons were identical in size in CtBP1-KO (30±0.3 nm, mean±standard error, n=117, rod ribbons; 31±0.3 nm, mean±standard error, n=249, bipolar-cell ribbons) and WT ribbon synapses (30±0.3 nm, n=118, rod ribbons; 31±0.3 nm, n=235, bipolar-cell ribbons), which suggests that CtBP1 does not influence the formation of synaptic vesicles. This is consistent with the finding that synaptic vesicle recycling in mouse bipolar neurons primarily depends on clathrin-mediated endocytosis and dynamin [20], whereas CtBP1 is thought to control membrane fission in pathways that are independent of dynamin and clathrin [23]. However, in CtBP1 null retina, we did observe a small but statistically significant reduction in the overall density of free synaptic vesicles in the cytoplasm of terminals in both rod photoreceptors (WT, 859±32 vesicles/μm^2^, mean±standard error, n=12 terminals from two animals; KO, 722±44 vesicles/μm^2^, mean±standard error, n=15 terminals from two animals; p=0.02) and bipolar neurons (WT, 321±15 vesicles/µm^2^, n=20 terminals from two animals; KO, 258±14 vesicles/µm^2^, n=19 terminals from two animals; p=0.004). This CtBP1-dependent reduction in number of vesicles may reflect a minor contribution of nonclathrin-mediated endocytosis to vesicle recycling in mammalian bipolar cells and rod photoreceptor cells, possibly similar to the bulk retrieval shown to be a major contributor in fish bipolar neurons [24,25].

### CtBP1 is not required for vesicle turnover at ribbon synapses

**Figure 7:**
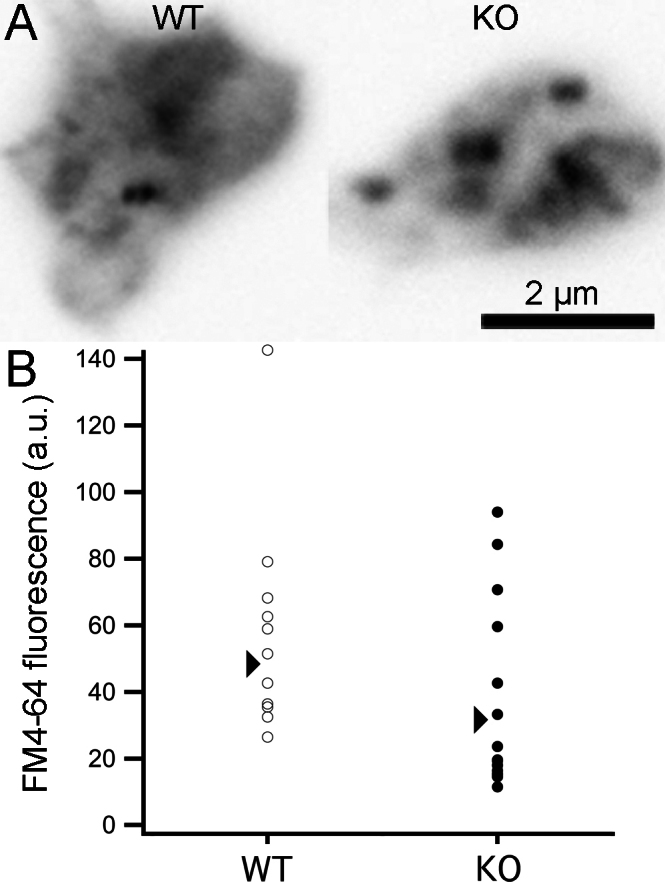
Bipolar cells from C-terminal-binding protein 1(CtBP1)-knockout retina loaded normally with FM4-64. **A**: The images show a single confocal optical section of FM4-64 fluorescence in a live mouse bipolar cell terminal from wild-type (WT) or CtBP1-knockout (KO) retina. Cells were loaded with FM4–64 in the presence of high external [K^+^] to activate calcium channels and trigger synaptic vesicle cycling, followed by washing with Advasep-7 to rapidly remove plasma-membrane dye. **B**: Each data point represents results from a single WT terminal (open circles; n=11 cells in three animals) or KO terminal (filled circles; n=16 cells in three animals). The arrows indicate the average FM4–64 fluorescence for each genotype.

Because there was a small decrease in the cytoplasmic density of free vesicles in CtBP1-KO synapses, we next examined whether synaptic vesicle recycling is impeded significantly in CtBP1-KO bipolar neurons. We compared the activity-dependent loading of the styryl dye, FM4–64, in isolated bipolar cells from WT and CtBP1-KO animals as an index of endocytosis efficiency [10,26,27]. Bipolar cells were incubated with FM4–64 in the presence of high-K^+^ external solution (see Methods) to induce exocytosis, followed by compensatory endocytosis and trapping the dye in internalized vesicles; cells were then washed thoroughly with solution containing Advasep-7 to remove noninternalized dye [10,20]. Figure 7A shows examples of terminals containing internalized FM4–64, and Figure 7B shows that there is no statistically significant difference between the two genotypes in fluorescence intensity (WT, 57.9±9.8 a.u./μm^2^, mean±standard error, n=11 terminals in three animals; KO, 34.6±6.8 a.u./μm^2^, mean± tandard error, n=12 terminals in three animals; p=0.07), indicating that activity-dependent loading of dye was largely unaffected by the loss of CtBP1. We interpret these results to indicate that CtBP1 is not a primary regulator of compensatory endocytosis at ribbon synapses.

**Figure 8:**
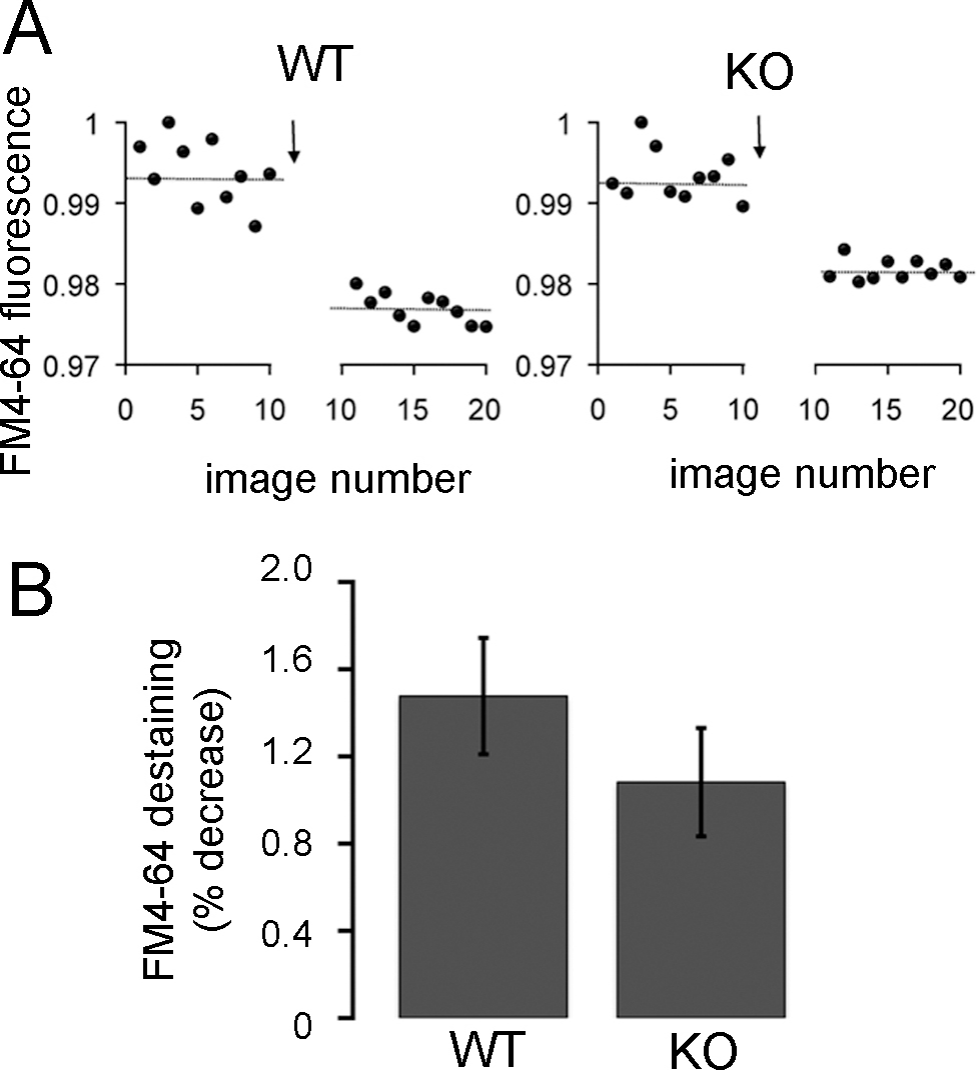
Absence of C-terminal-binding protein 1(CtBP1) does not affect FM4–64 destaining evoked by depolarization in bipolar-cell synaptic terminals. **A**: The graphs show examples of destaining elicited by activation of calcium current for 500 ms (arrow) in a wild-type (WT) and CtBP1-knockout (KO) bipolar-cell terminal, preloaded with FM4–64. Each data point shows the average fluorescence of the terminal for each image in a series of ten confocal images before and ten images after depolarization. The horizontal lines indicate the average fluorescence for each series. Fluorescence is normalized with respect to the maximum. An arbitrary break was introduced in the abscissa for visual clarity. **B**: The graph shows the average percentage loss of FM4–64 fluorescence elicited by 500-ms depolarization to activate calcium current and trigger synaptic vesicle fusion in WT (n=14 cells in three animals) or KO (n=15 cells in three animals) bipolar cells. The error bars show ±one standard error of the mean. The difference is not statistically significant (p=0.30).

**Figure 9:**
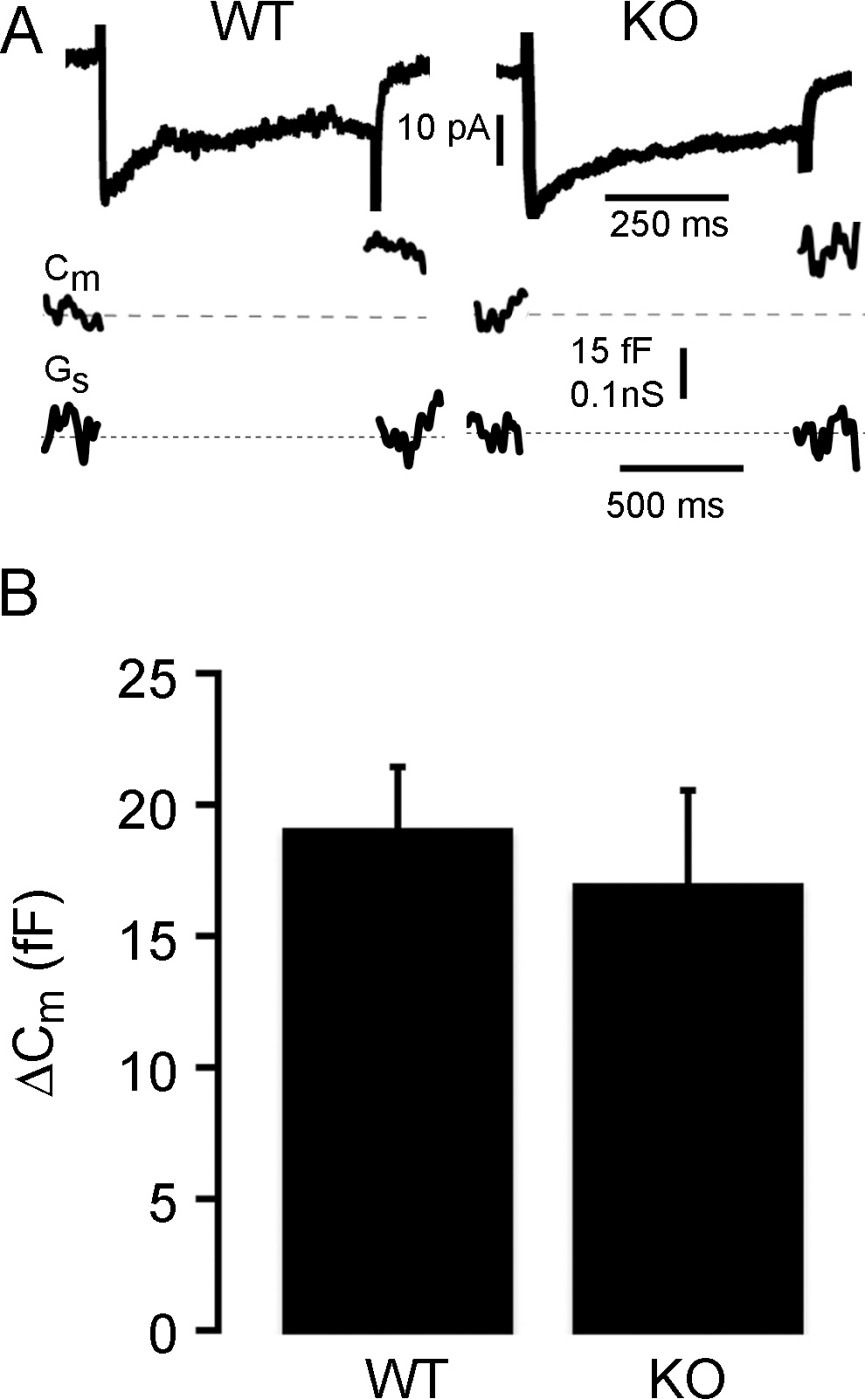
Absence of C-terminal-binding protein 1(CtBP1) does not affect depolarization-evoked exocytosis measured by changes in membrane capacitance. **A**: The traces show examples of calcium current (upper traces), the change in membrane capacitance (Cm, middle traces), and change in series conductance (Gs, lower traces) evoked by 500-ms depolarization from 0 to −65 mV in wild-type (WT) and CtBP1-knockout (KO) bipolar cells. **B**: The graph shows the average change in membrane capacitance, ΔCm, elicited by 500-ms depolarization in WT (n=22 cells in four animals) or KO (n=19 cells in four animals) bipolar-cell terminals. The error bars show the standard error of the mean for each group. The difference is not statistically significant (p=0.54).

CtBP1 has also been suggested to facilitate exocytosis at the ribbon [2], possibly by serving as a lysophosphatidic acyl-coenzyme A transferase (LPAAT) to modulate the curvature of lipid membranes [28]. To check if exocytosis is affected in CtBP1-KO mouse bipolar cells, we triggered bouts of exocytosis by activating calcium current with 500 ms depolarization in terminals previously loaded with FM4–64 and measured the resulting loss of trapped dye as vesicles fused (Figure 8A). As shown in Figure 8B, there was no significant difference in the percent of destaining produced by depolarization in WT and KO bipolar cell terminals. Calcium currents were also similar in the two genotypes, as illustrated by the examples shown in the upper traces of Figure 9A, and neither the peak amplitude of the calcium current (WT, 15.6±2.2 pA, mean±standard error, n=9 cells in four animals; KO, 12.0±2.1 pA, mean±standard error, n=9 cells in four animals; p=0.31) nor the total charge transferred during a 500-ms depolarization (WT, 7.5±0.8 pC, n=9 cells in four animals; KO, 7.1±1.0 pC, n=9 cells in four animals; p=0.73) was significantly different. To corroborate the findings based on FM4–64 destaining, we also measured membrane capacitance changes (see examples in Figure 9A, middle traces) as an index of synaptic vesicle fusion in response to a 500-ms depolarization, and once again there was no significant difference between WT and CtBP1-KO bipolar cells (Figure 9B). Therefore, we conclude that exocytosis triggered by calcium influx is unaffected by the absence of CtBP1 in synaptic terminals of bipolar neurons.

After depletion of the releasable pool of vesicles, the pool is refilled from reserve vesicles, which requires approximately 20 s for completion (e.g., [29]). To determine if pool refilling in CtBP1-KO bipolar cells is comparable to WT bipolar cells, we measured capacitance responses to pairs of 500-ms depolarizations separated by 30 s, which is sufficient for recovery of evoked exocytosis. There was no change in calcium current evoked by the two pulses in either WT (second current 116±12% of first; mean±standard error, n=16 cells in four animals) or CtBP1-KO bipolar cells (second current 113±7% of first; n=23 cells in four animals), so inactivation of calcium channels was not a concern. In WT bipolar cells (n=21 cells in four animals), the second bout of exocytosis averaged 85±20% of the first, while in CtBP1-KO bipolar cells (n=18 cells in four animals), the second response was 88±13% of the first. Therefore, we found no evidence of any functional deficits in either exocytosis or pool refilling in the absence of CtBP1.

## Discussion

Retinal ribbon synapses did not exhibit any striking differences in mice lacking CtBP1, with the exception of a small decrease in the number of cytoplasmic reserve vesicles in both photoreceptor and bipolar-cell synaptic terminals. In keeping with the structural integrity of the synapses, bipolar cells isolated from CtBP1-KO mice had no detectable functional deficits in synaptic vesicle cycling monitored by FM4–64 uptake and destaining and by changes in membrane capacitance. This suggests that CtBP1 is not essential for structural assembly of ribbons, neurotransmitter release, or vesicle recycling. So, if CtBP1 is a component of ribbons [2], why does its absence in CtBP1-KO mice have no effect on ribbon function in our experiments? CtBP1 and CtBP2 form homodimers and heterodimers that are important in their roles in regulation of gene transcription [3,4]. This raises the possibility that cytoplasmic CtBP1 is recruited to ribbons by forming dimers with the B domain of RIBEYE, but the dimerization is an epiphenomenon in this context. Therefore in this view, loss of CtBP1 would have no functional consequence for ribbon synaptic function. Alternatively, recruitment of CtBP1 to the ribbon may be functionally significant, but CtBP2 may compensate for the absence of CtBP1 at ribbons in CtBP1-KO mice just as CtBP2 is thought to substitute for CtBP1 in the developmental regulation of gene transcription in CtBP1 null mice [9,30]. Although CtBP2 had been viewed as an exclusively nuclear protein, it has recently been demonstrated to be localized in other cellular compartments, including strong expression at a subset of conventional synapses in the central nervous system [16]. Therefore, cytoplasmic CtBP2 could form homodimers with the B domain of RIBEYE to functionally replace the CtBP1–RIBEYE heterodimer. We did observe an increase in anti-CtBP2 staining at ribbons in CtBP1 null mice (Figure 3), which would be consistent with a compensatory increase in CtBP2 in the absence of CtBP1. However, other interpretations of the increased immunostaining are possible. Without a clear functional or structural phenotype as shown here, the CtBP1-KO mouse is not well suited to resolve this issue, and further work to acutely disrupt CtBP1 expression will be required.
